# Characteristics of Motor Resonance Predict the Pattern of Flash-Lag Effects for Biological Motion

**DOI:** 10.1371/journal.pone.0008258

**Published:** 2010-01-07

**Authors:** Klaus Kessler, Lucy Gordon, Kari Cessford, Martin Lages

**Affiliations:** 1 Department of Psychology, University of Glasgow, Glasgow, United Kingdom; 2 Centre for Cognitive Neuroimaging (CCNi), Department of Psychology, University of Glasgow, Glasgow, United Kingdom; Ecole Polytechnique Federale de Lausanne, Switzerland

## Abstract

**Background:**

When a moving stimulus and a briefly flashed static stimulus are physically aligned in space the static stimulus is perceived as lagging behind the moving stimulus. This vastly replicated phenomenon is known as the Flash-Lag Effect (FLE). For the first time we employed biological motion as the moving stimulus, which is important for two reasons. Firstly, biological motion is processed by visual as well as somatosensory brain areas, which makes it a prime candidate for elucidating the interplay between the two systems with respect to the FLE. Secondly, discussions about the mechanisms of the FLE tend to recur to evolutionary arguments, while most studies employ highly artificial stimuli with constant velocities.

**Methodology/Principal Finding:**

Since biological motion is ecologically valid it follows complex patterns with changing velocity. We therefore compared biological to symbolic motion with the same acceleration profile. Our results with 16 observers revealed a qualitatively different pattern for biological compared to symbolic motion and this pattern was predicted by the characteristics of motor resonance: The amount of anticipatory processing of perceived actions based on the induced perspective and agency modulated the FLE.

**Conclusions/Significance:**

Our study provides first evidence for an FLE with non-linear motion in general and with biological motion in particular. Our results suggest that predictive coding within the sensorimotor system alone cannot explain the FLE. Our findings are compatible with visual prediction (Nijhawan, 2008) which assumes that extrapolated motion representations within the visual system generate the FLE. These representations are modulated by sudden visual input (e.g. offset signals) or by input from other systems (e.g. sensorimotor) that can boost or attenuate overshooting representations in accordance with biased neural competition (Desimone & Duncan, 1995).

## Introduction

### The Flash-Lag Effect (FLE)

When a moving stimulus and a briefly flashed static stimulus are shown spatially aligned the static stimulus is perceived as lagging behind the moving stimulus. This vastly replicated and extended phenomenon is called the Flash-Lag Effect (FLE) [1]. The FLE has received a great deal of attention because it initiated a fundamental discussion about the properties of the visual system: Does the early (low level) visual system already compensate for neural delays in terms of visual prediction as proposed by Nijhawan [1], [2] or is such a predictive mechanism only observed within the sensorimotor system? The predictive mechanisms of the motor system are widely accepted since without compensation for neural delays motor actions in response to dynamic events would always lag behind [2, for review]. For example, any sport involving perception of- and action to high speed movements would be impossible to perform. For a comprehensive overview of this debate see Nijhawan's [2] recent article in Behavioural and Brain Sciences and the associated peer discussion. In the following we summarize some of the key arguments of this debate to provide a background for our study.

Two conditions are crucial for the understanding of the FLE, namely the “flash-initiated” and “flash-terminated” condition. In the latter condition a stimulus moves on screen before it disappears together with the flashed stimulus. Here the FLE vanishes completely, which imposes a serious problem for the visual prediction (VP) account. If the FLE were due to extrapolation of the motion trajectory then this should lead to an “overshoot” even if the stimulus disappears. Nijhawan [2] proposed a competition (along the lines of the “biased competition model” [3], [4]) between the extrapolated, overshooting representation and the ‘correct’ representation generated by the sudden offset of the stimulus. Due to the strength of the transient offset activation the latter representation is likely to win the competition thereby providing the veridical location of the stimulus [5]. An essential prediction of biased competition is that a weaker offset signal would favour the extrapolated motion representation. Recent findings indeed support this extended VP account by showing that fading out the moving stimulus, i.e. smoothing the sudden offset [6], placing the sudden offset into the retinal blind spot [7], as well as other manipulations that weaken the sudden offset (e.g. [8], [9]) promote the idea of overshooting representations in the visual system. Neurophysiological evidence for the extended VP hypothesis comes from studies in monkeys that show an FLE in V4 neurons in the flash-terminated condition [10] supporting the existence of high-level overshooting representations within the visual system. The notion of a competition between low-level offset signals and high-level overshooting representations is important for the purpose of our study, since biological motion has the potential to boost high-level overshooting motion representations possibly tipping the balance of the competition.

In the flash-initiated condition the flash is spatially aligned with the moving stimulus at the start of the trial. While the flash disappears the moving stimulus continues on its trajectory. Interestingly, this flash-initiated condition induces an FLE that can be even stronger than in the standard continued motion condition [11], [12]. Note that this type of FLE is related to the “Fröhlich Effect” [13] but differs from it because the moving stimulus is not solely misplaced in the direction of motion but in relation to the flashed static stimulus. The FLE in flash-initiated conditions suggests an early origin of the FLE within the visual system (see [2] for review) and together with an FLE in specific flash-terminated conditions [6] speaks against the idea that only the sensorimotor system uses predictive mechanisms to compensate for delays in visual processing. This latter view (e.g. [14]) is mainly based on the observation that participants who point to a moving stimulus that just disappeared systematically overshoot the actual stimulus position similar to the FLE in the continuous motion condition. It is therefore essential to explore the relation between the visual and the sensorimotor system in the context of the FLE.

Nijhawan and Kirschfeld [15] observed an FLE with limb action. That is, participants were moving their arm without being able to see it. When a flash was administered in perfect alignment with the (invisible) moving limb participants showed an FLE. This reveals the tight connection between the visual and the sensorimotor system and further suggests that the two systems employ similar (predictive) mechanisms [2]. Ichikawa and Masakura [16] manipulated voluntary control of the motion in their experiments: In one condition participants fully controlled the motion on the screen with a mouse connected to the PC. In this ‘full control’ condition as well as in a ‘partial control’ condition, where participants maintained an illusion of control, the FLE disappeared. This is an important finding as it suggests a complex interaction between the visual and the sensorimotor system: While both systems tend to overshoot when measured separately (cf. Nijhawan and Kirschfeld [19]), when combined they seem to cancel each other out and participants are able to accurately locate the stimulus.

In the work presented here we explore the relation between the visual and the sensorimotor system with respect to the FLE and predictive coding. Although biological motion is an obvious candidate in this context, it has not been employed in studies on the FLE so far. Firstly, biological motion is processed by visual as well as somatosensory brain areas, which makes it a prime candidate for investigating the interplay between the two systems. Secondly, it is of major interest because of its social and ecological relevance–the FLE debate tends to recur to evolutionary arguments (e.g. [17], [2], [18]), while most studies employ highly artificial stimuli with constant velocities to investigate the characteristics of the FLE thereby assuming that results will generalize to non-linear “real-world” situations. In the following we will give a brief summary of the relevant biological motion literature to motivate the specific design of our study.

### Biological Motion

Humans have evolved to optimally cope with dynamic events in a social environment. At a basic level this implies the ability to decode others' movements, quickly understand the goals behind their actions and anticipate their ‘next move’ (e.g. [19]–[23]). This makes the perception of biological motion special compared to other moving stimuli. The ability to imitate others has been a very important step in the evolution of mankind suggesting a close link between visual perception of the movements of others and our own actions (e.g. [24], [25]). The special status of biological motion has been demonstrated in various experimental settings, showing, for example, that reaction times to biological cues are faster than to non-biological spatial cues [26]–[28], and that imitation of biological movements is faster than kinematically identical motion of non-biological stimuli [29]–[34]. Accordingly, it has been suggested that during imitation, the observer transforms the visual input of a motor act provided by a peer model, into a corresponding motor output [25]. This is the so-called “Action Observation Execution Matching” hypothesis - or more generally “motor resonance” (e.g. [35]) which implies that a match between an observed movement and the observer's motor repertoire directly subserves the decoding of biological motion.

The mirror neuron system (MNS) in ventral premotor and inferior parietal areas has been proposed as the neural substrate for motor resonance [36], [37], [35], [38]. The MNS interacts closely with perceptual areas in the superior temporal and occipito-temporal cortex [39], [40], [32], [33], [41] as well as large parts of the motor system [33], [42]–[45]. This makes biological motion a prime candidate for studying visual and sensorimotor interactions during the FLE. The notion of visual prediction and competition between neural representations at various levels (i.e., between a strong bottom-up offset signal and a central overshooting representation), offers a fitting framework for conceptualizing the influence of motor resonance on the visual system. Three processing characteristics of the MNS are relevant to this issue: anticipation, agency, and perspective. They allow for specific hypotheses regarding the pattern of FLE as revealed with biological motion.

**Figure 1:**
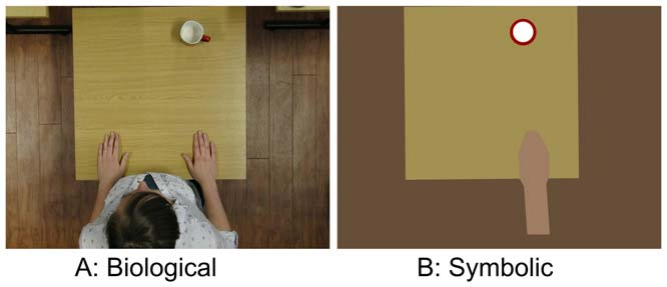
Stimulus type. Start positions for the biological (A) and the symbolic (B) clips in the 1^st^ person perspective (cf. Fig. 3).

**Figure 2:**
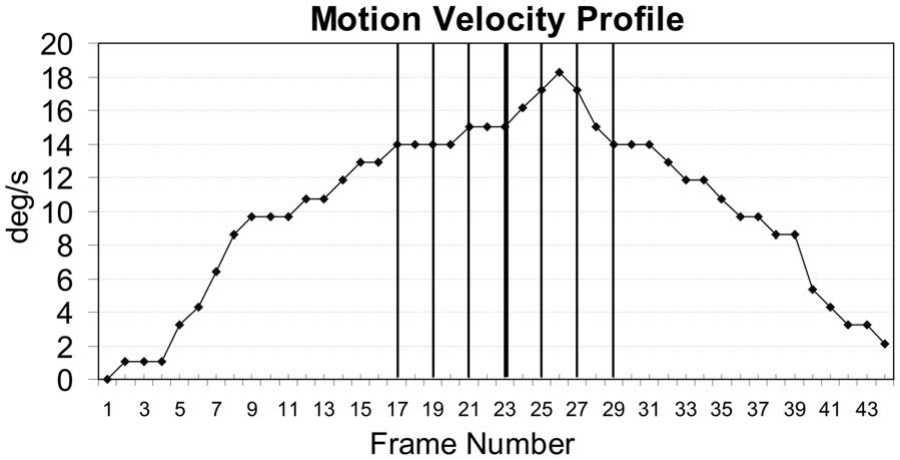
Motion velocity profile. Velocity is measured in degrees per second (deg/s) and is plotted for each of the 44 movie frames. A constant velocity as usually employed in FLE research would be represented by a line parallel to the x-axis. The depicted profile was derived from the recorded ‘real’ biological movement (i.e. a person reaching for a cup in top-view, cf. Fig. 3) and it was identical for the biological and the symbolic motion conditions in the experiment. Note that this profile only represents the task-relevant motion in the vertical direction (cf. Fig. 3). Vertical lines in the graph denote the frames in which the flash could occur – the thicker line is lag 0 (cf. Fig. 3). In the flash-terminated trials the motion was aborted after the respective frame with the flash. Further explanations in the text.

It has been suggested that the MNS is a predictive system [19], [46], [21], [22] that *anticipates* movements and extrapolates the observed action towards its goal. In the present study we employed goal-directed biological motion, i.e., a person reaching for a mug, in order to generate the maximum possible resonance with the observer's MNS. The predictive characteristics of the MNS allows for the following hypotheses regarding the FLE. If we assume that its predictive characteristics boost motion extrapolation than we would expect a stronger FLE for biological than for symbolic motion (Fig. 1), even if the same acceleration profile is employed in both conditions (Fig. 2). This should be the case for the standard continuous motion conditions, yet, it might be even possible to obtain an FLE in flash-terminated conditions where the MNS may boost an overshooting motion representation.

Neural activation in the MNS is not only more robust for goal-directed actions but also for movements that are imitated rather than passively observed. The amount of experienced *agency* is therefore another relevant factor that modulates the amount of resonance between the observed movement and the observer's motor repertoire. Ramachandran and colleagues [47], [48] reported that resonance in the MNS can induce an illusion of voluntary control and agency if the observed effectors are spatially compatible with the observer's limbs. However, Ichikawa and Masakura [16] reported that agency in form of voluntary motion control–or the illusion of it - obliterates the FLE. It could therefore be that movement imitation induces an illusion of voluntary control which actually works against motion extrapolation in the visual system and attenuates the FLE. Alternatively, the illusion of voluntary control with biological motion may have an opposite effect compared to the illusion of voluntary control with symbolic stimuli. While control of symbolic motion seems to diminish the FLE (cf. [16]), enhanced predictive motor resonance in the MNS could lead to a stronger FLE with biological motion. Nijhawan and Kirschfeld's report of a movement-induced FLE [15] could point into this direction. It is therefore difficult to predict the direction of an effect induced by tracking/imitation of stimulus motion (i.e. enhancing or reducing the FLE). However, we expected a differential effect for tracking/imitation vs. passive observation as well as a different pattern for biological compared to symbolic motion.

**Figure 3:**
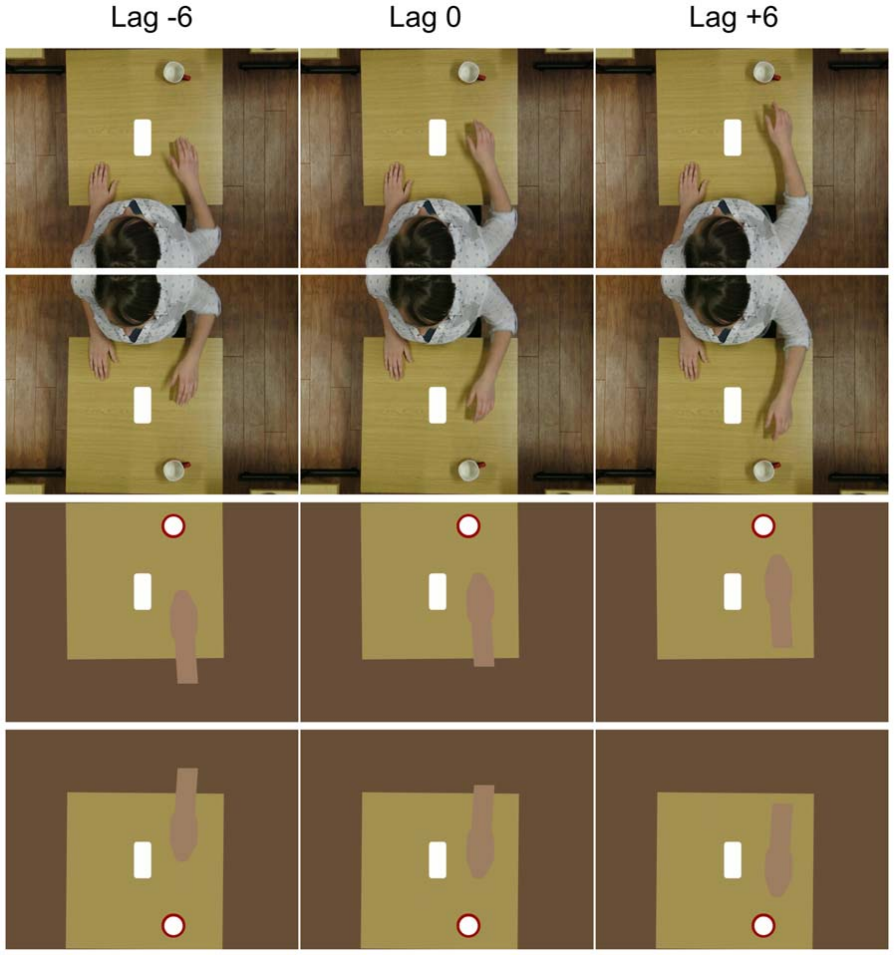
The flash and the lags. The Figure shows example stimuli for lags of −6, 0, and +6 frames (columns) for the biological (rows 1 and 2) and the symbolic (rows 3 and 4) stimuli and for the 1^st^ person (rows 1 and 3) and the 3^rd^ person (rows 2 and 4) perspective. In the 1^st^ person perspective always the right hand of the actor moved, while in the (mirrored) 3^rd^ person perspective always the left hand moved in order to optimise resonance with the right hand of the observer during tracking. Further explanations in the text.

Finally, Jackson et al. [49] reported that imitation was more quickly initiated with a movement shown from a *1st person perspective* compared to a *3rd person perspective* (Fig. 3). The authors also found that the 1st person and 3rd person perspectives relied on slightly different neural activations and that there was increased activity in the sensorimotor system for the 1st person perspective compared to the 3rd person perspective. This may indicate an enhanced dissociation between vision and proprioception in the 3rd person perspective. These findings strongly suggest that perspective has an effect on biological motion processing within the MNS, with the 1st person perspective facilitating resonance. For our present study this provided the following predictions. For biological motion the 1st person perspective generates the strongest resonance and should therefore induce the strongest FLE. However, this should depend on the factor ‘agency’. In the 1st person perspective an illusion of control should be maximal when stimulus motion on screen and the movement of the observer are aligned, whereas in the 3rd person perspective the motion on screen is perceived as seen through a mirror. We described above that an illusion of control could either reduce or enhance the FLE for biological motion, yet, in both cases this would lead to an interaction between perspective and agency for biological stimuli. For symbolic motion there is no perspective overlap between actor and observer so no such interaction was expected.

Resuming our predictions for the flash-terminated condition, we expected an increased FLE for the condition that generates the strongest overshooting representation, i.e. the strongest predictive motor resonance within the MNS. This is clearly the case for biological motion in the 1st person perspective, yet, as pointed out, possibly in only one of the agency conditions–which exactly, depends on whether an illusion of control is induced and whether illusionary control of a biological motion attenuates or boosts the FLE.

## Methods

### Ethical Statement

Ethical approval was formally obtained from the Faculty Ethics Committee (FEC) of the Faculty for Mathematical and Information Sciences (FIMS https://web1.psy.gla.ac.uk/ethics/). All participants gave written consent and the experimental procedures complied with the Declaration of Helsinki.

### Participants

16 right-handed volunteers (5 female), aged between 19–28, participated in this experiment. Four participants were replaced because data were noisy and data fits did not meet a preset precision criterion (see details below).

### Stimuli & Apparatus

The original movement was recorded with a Casio EX-F1 Exilim digital camera, capturing the movement at a rate of sixty frames per second (60 Hertz) with high resolution. The basic stimuli were movie clips consisting of 44 single frames (1400×1050 pixels at a duration of 16.7 ms per frame). Each sequence of images was projected by a high-resolution data projector (JVC D-ILA SX21) at 60 Hz (non-interleaved) on a high contrast gain projection screen (Harkness, 2 m by 1.5 m) from a viewing distance of 4 m in a dark room (visual angle of about 26.5°×20.5°) in order to approximate the natural size of the recorded scene. The experiment was programmed with E-prime® 2.0 and run on a 3 GHz Dual Core, 3 GB RAM PC with a 256 MB ATI Radeon 2400 XT graphics card.

In the biological condition each clip showed in top view a person sitting at a table and reaching for a cup in front of them (see Fig. 1A). For the symbolic clips the original footage was edited frame by frame replacing the floor, the table, and the cup by simple geometric shapes matching in color but without textures and shadows (Fig. 1B). The person in the clip was omitted; only the (moving) right lower arm was replaced frame by frame with a shape that approximated the outline of the hand and lower arm in the original footage (Fig. 1B; compare Fig. 3). This ensured that the symbolic shape had corresponding motion parameters as the hand (see Fig. 2). Most importantly, the contours in the direction of movement (e.g. fingertips) were perfectly aligned for each frame and each clip of the biological and the symbolic conditions.

The original footage was edited to insert a white rectangle next to the hand to induce a flash. The flash was presented in different frames so that the movement could be either behind (−6, −4, −2 frames), at the same position (±0 frames) or ahead (+2, +4, +6 frames) of the flash (see Figure 2). The flash always appeared for one frame only (16.7 ms) at the same location near the middle of the table. Note that if we would have varied the position of the flash then the table could have provided a frame of reference, hence, possibly influencing the observers' judgements. The flashed stimulus was perfectly aligned (neither ahead nor behind) with the tip of the moving stimulus in the 0 lag condition at frame 23, as shown in Figure 2 (middle column). At other lags–where the moving stimulus was either ahead of the flash, or lagging behind (see Figure 2)–the flash was presented in steps of 2 frames before or after frame 23 (lag −6 = frame 17; lag −4 = frame 19; lag −2 = frame 21; lag 0 = frame 23; lag +2 = frame 25; lag +4 = frame 27; lag +6 = frame 29).

In addition to the type of stimulus (biological vs. symbolic) we also varied the perspective. In Figure 2, row 1, the observer is aligned with the left and right of the depicted actor (the observer's right is the actor's right) so the movement is perceived from a 1^st^ person perspective (cf. [49]). We also employed a mirrored version of the images as shown in Figure 2 (2^nd^ row) where the observer and the depicted person were positioned opposite to each other (i.e., the observer's right was the person's left) so the movement was perceived from a mirrored or 3^rd^ person perspective (cf. [49]). The images in the symbolic condition were varied accordingly (Figure 2, rows 3 and 4) but since there was no actor no alignment or dissociation in perspective should occur.

We also employed a tracking condition to enhance the degree of agency that participants experience. In one block of trials observers passively watched the stimuli on the projection screen whereas in another block they were instructed to track the movement with their right hand. Biological stimuli were designed to match with the observer's right hand by showing a right hand movement in the 1^st^ person perspective and a left hand movement in the mirrored 3^rd^ person perspective (see Figure 2) conform to findings by Jackson et al [49] and Koski et al. [50] regarding optimum motor resonance in the MNS (see [51] for a discussion). In the symbolic condition no motor resonance is expected to occur, but equivalent to Ichikawa and Masakura's [16] findings in their ‘no control’ condition a stronger FLE should be observed for a concurring arm movement with no control over the visual motion.

### Procedure & Design

Each trial started with the presentation of a white fixation cross on a grey background for 100 ms. Then the static image of the first frame or starting position was shown for 1000 ms (see Figure 1), before movement commenced. In the continuous motion condition each clip lasted 733 ms (44 single frames at a duration of 16.7 ms). In the flash-terminated trials a uniform gray background screen was shown immediately after the frame with the flash. At the end of each trial participants indicated by means of alternative button presses whether they had perceived the moving stimulus ahead or behind the flash. Participants always responded with their left hand.

The trials were grouped into 4 blocks depending on the stimulus type of the shown movement (biological vs. symbolic) and on agency (tracking vs. no-tracking). This was to avoid carry-over effects [e.g. 52] between no-tracking trials and tracking trials and between symbolic trials and biological trials, respectively. The 2 biological blocks and the 2 symbolic blocks were administered on two separate days to further reduce carry-over effects, with each session lasting about 2 hours. The block order within and between days was permutated across participants with 4 participants in each block sequence (Sequence 1: day1, block1 = biological, tracking; block2 = biological no-tracking; day2, block1: symbolic, tracking; block2: symbolic no-tracking. Sequence 2: day1, block1 = biological, no-tracking; block2 = biological tracking; day2, block1: symbolic, no-tracking; block2: symbolic tracking. Sequence 3: day1, block1: symbolic, tracking; block2: symbolic no-tracking; day2, block1 = biological, tracking; block2 = biological no-tracking. Sequence 4: day1, block1: symbolic, no-tracking; block2: symbolic tracking; day2, block1 = biological, no-tracking; block2 = biological tracking.). Each experimental block started with 13 practice trials followed by 420 experimental trials. A break was administered after every 140 trials. The total number of trials was 1680.

We employed a four-way repeated measures design for statistical analysis (GLM module for repeated measures in Statistica®). The factors were stimulus type (biological vs. symbolic), perspective (1^st^ vs 3^rd^ person), agency (tracking/imitation vs. no-tracking/observation), and motion type (continuous vs. flash-terminated). In addition we also employed 7 different lags of the moving stimulus in relation to the flash for each factor combination (stimtype (2) x tracking (2) x perspective (2) x motion (2)). Each combination and lag was repeated in 15 trials.

### Psychometric functions

**Figure 4:**
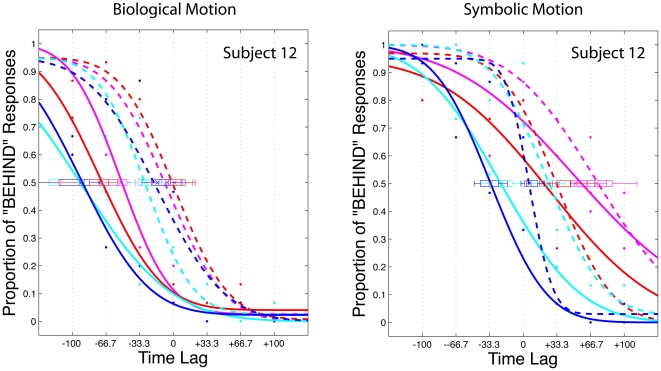
Psychometric functions for subject 12. (A) Biological Motion (B) Symbolic motion. Each graph shows the frequency of “behind” responses (y axis) against the flash lag (ms; x axis). The individual data points are fitted by cumulative Gaussian psychometric functions for each of the 8 biological (A) and 8 symbolic (B) motion conditions. The box and error bar at each 50% point (PSE) indicate 95% and 99% Confidence Interval respectively. The steepness of the curve, expressed as the SD, gives an indication of the discrimination performance (or JND). Solid line: continued motion, dashed line: flash-terminated motion. Magenta: “1st person no tracking” condition; Red: “3rd person no tracking” condition; Cyan: “1st person tracking” condition Blue: “3rd person tracking” condition; Further explanations in the text.

A cumulative Gaussian was fitted to the data of each subject and condition across the 7 lags [53], [54] suggesting a decrease of responses for flash perceived “behind” or equivalently an increase of responses for flash perceived “ahead” of the moving stimulus. “Point of Subjective Equality” (PSE; 50% point or mean) and the standard deviation (SD) were estimated for each of the participants and conditions as a measure of accuracy and precision of the FLE, respectively. A negative value suggests a PSE before lag 0, indicating that an FLE did occur. That is, “ahead” judgments dominate while the movement is actually lagging behind the flash. Positive values would actually indicate a “Flash-Lead Effect” (FLeadE) where “behind” judgments persist beyond lag 0 although the movement is ahead of the flash. In fact we expected this latter effect in the flash-terminated symbolic condition due to the nature of our stimuli: At lag 0 (see Figure 3) only the tip of the moving stimulus (hand/symbolic shape) is aligned with the broader front of the high-contrast flash, thus, it may induce the illusion in the flash-terminated stimuli (see static images at lag 0 in Fig. 3) that the hand/symbolic shape is lagging behind the flash. This does not affect the outcomes of the experiment since we were interested in the pattern-differences between the biological and the symbolic conditions depending on agency and perspective, and not in an absolute measure of the FLE. As a typical example, the psychometric functions of Subject 12 are reported in Figure 4. Four participants had to be replaced because discrimination performance as expressed by the SD of the fitted cumulative Gaussian was greater than 16 frames in one or more conditions.

## Results

**Figure 5:**
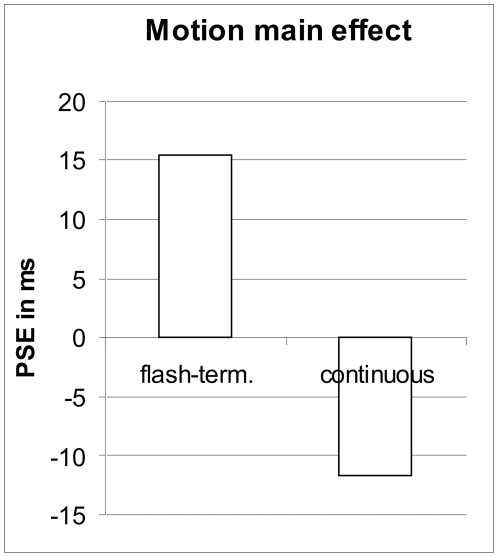
Motion main effect. Negative values indicate an FLE in the continuous motion condition while positive values indicate a ‘flash-lead effect’ (FleadE) in the flash-terminated condition. Further explanations in the text.

We conducted a repeated measures ANOVA with the 4 factors stimulus type (biological vs. symbolic), perspective (1^st^ vs 3^rd^ person), agency (tracking/imitation vs. no-tracking/observation), and motion type (continuous vs. flash-terminated). The average and standard deviation for each condition are shown in Table 1. The main effect of motion type reached significance (F(1,15) = 5.5, *p*<.04, *η^2^_p_* = .269) with continuous motion trials showing an FLE of −11.7 ms on average, while flash-terminated trials did not (Fig. 5). In the latter we observed a flash-lead effect (FLeadE) of +15.4 ms due to the nature of our stimuli (Fig. 3; see Methods for details). The difference of 27.1 ms between the two conditions lies within the range of a classical FLE measured at a mean velocity of 10–12 deg/s [23].

**Table 1:** PSE means and standard deviations (stdev) for each condition.

	*Biological*							
	NoTracking				Tracking			
	1st person perspective		3rd person perspective		1st person perspective		3rd person perspective	
	continuous	flash-stopped	continuous	flash- stopped	continuous	flash- stopped	continuous	flash- stopped
mean PSE	**−21.92**	**1.05**	**−4.78**	**12.06**	**−13.64**	**12.26**	**−29.12**	**19.38**
stdev	**69.96**	**26.94**	**64.83**	**28.30**	**75.62**	**48.61**	**75.45**	**35.15**
	*Symbolic*							
	NoTracking				Tracking			
	1st person perspective		3rd person perspective		1st person perspective		3rd person perspective	
	continuous	flash- stopped	continuous	flash- stopped	continuous	flash- stopped	continuous	flash- stopped
mean PSE	**−4.10**	**21.07**	**4.17**	**22.67**	**−20.22**	**11.86**	**−3.86**	**22.48**
stdev	**79.13**	**41.52**	**65.06**	**40.45**	**87.26**	**55.50**	**71.51**	**30.31**

**Figure 6:**
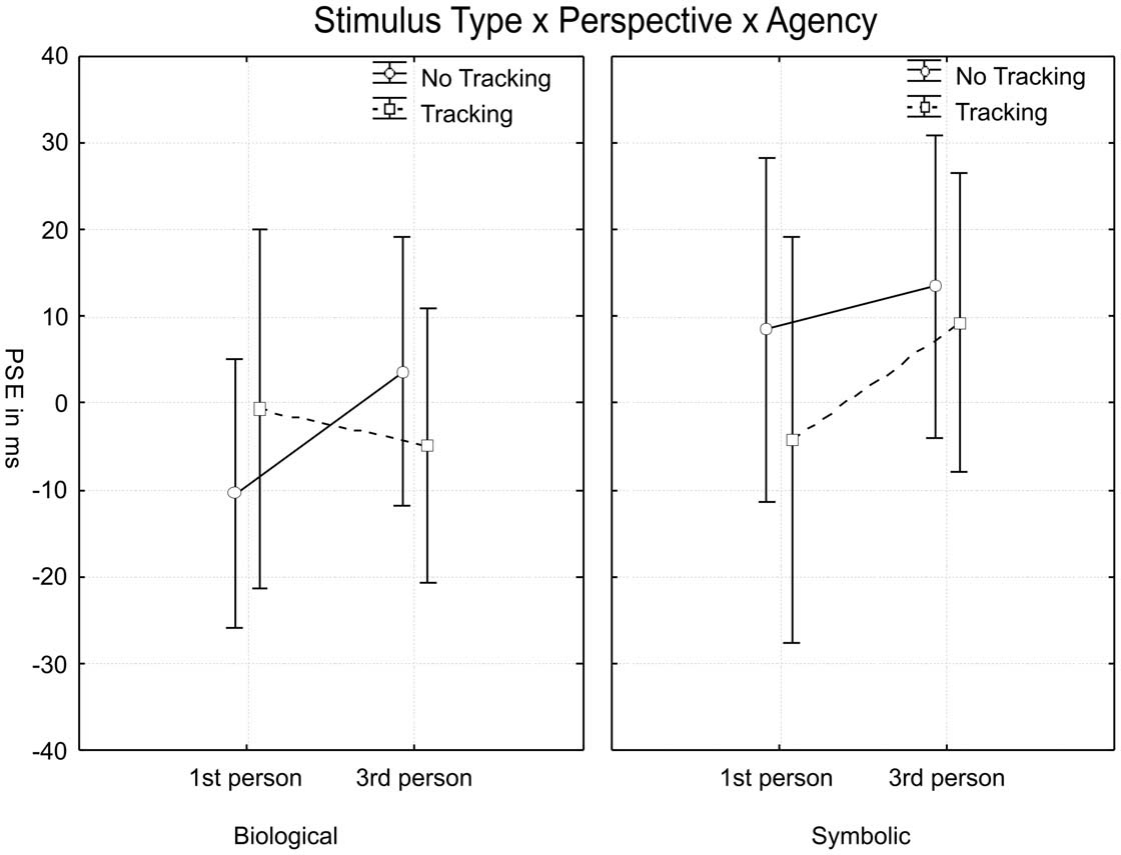
Interaction between stimulus type, perspective, and agency. The y-axis expresses the PSE in ms (compare Fig. 4). Biological motion is shown in the left and symbolic in the right graph. Vertical bars denote the 95% confidence interval. Further explanations in the text.

The interaction between stimulus type, perspective, and agency also reached significance (F(1,15) = 5.1, *p*<.04, *η^2^_p_* = .254). As shown in Figure 6 the FLE pattern for biological stimuli (left graph) was very different from the pattern observed for symbolic stimuli (right graph). This was confirmed by two separate ANOVAs for each stimulus type. For symbolic stimuli only the main effect of motion type reached significance (F(1,15) = 5.0, *p*<.05, *η^2^_p_* = .250) while for biological stimuli the interaction between perspective and agency was significant (F(1,15) = 5.7, *p*<.03, *η^2^_p_* = .276) in addition to the main effect of motion type (F(1,15) = 4.9, *p*<.05, *η^2^_p_* = .248). This confirmed our main prediction that we would observe a different FLE pattern for biological than for symbolic motion depending on perspective and agency. Specifically, we did predict an interaction between perspective and agency for biological but not for symbolic motion. As described in the Introduction the biological 1^st^ person perspective should generate the strongest motor resonance with the observer's MNS, yet, may also induce the strongest illusion of voluntary control during imitation (cf. [47], [48]), potentially leading to an obliteration of the FLE (cf. [16]). The latter seems to be the case: the FLE in the 1^st^ person perspective is attenuated during imitation compared to passive observation. The pattern is reversed for the 3^rd^ person perspective where an illusion of control is harder to establish, since motion on the screen and the observer's movement follow mirrored trajectories.

**Figure 7:**
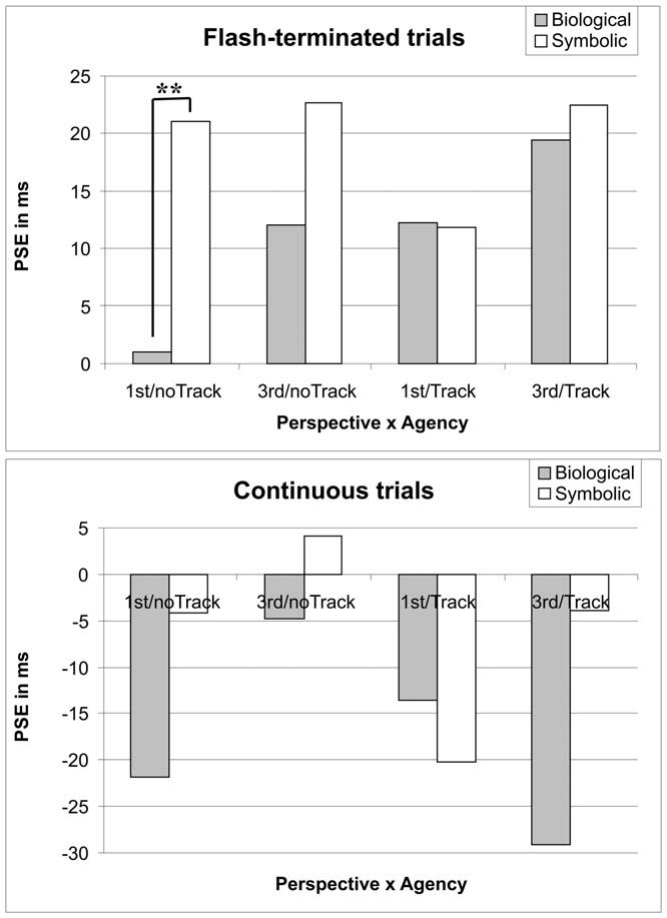
Flash-terminated and continuous trials. The x-axis shows the four combinations of perspective (1^st^ vs. 3^rd^ person) and agency (tracking vs. no tracking). The y-axis expresses the PSE in ms. A) The positive values in the flash-terminated trials reveal that we did not observe an FLagE (negative values) but a flash-lead effect (FLeadE, positive values) in the flash-terminated conditions due to the nature of our stimuli (see Methods). However, the condition that generates the strongest motor resonance (biological, 1^st^ person perspective), yet without the illusion of control (no tracking) shows the smallest FLeadE (i.e., more of an FLE than the other conditions). Two asterisks indicate p<.01. B) Predominantly negative values are observed in the continuous motion conditions. Further explanations in the text.

Taking this line of thought one step further, we did also predict that the condition that would generate the strongest motor resonance in the observer's MNS, yet, without inducing an illusion of control at the same time would be the best candidate to reveal an FLE - even in the flash-terminated condition. This was predicted for biological motion in the 1^st^ person perspective, while agency was expected to have an impact as well. As we have established above, it seems likely that an illusion of control is induced when tracking/imitation is employed in the 1^st^ person perspective (cf. [47], [48]) and that its effect is conform to Ichikawa and Masakura's findings, namely an attenuation of the FLE. Therefore the most likely condition to generate an FLE in flash-terminated trials was the biological, 1^st^ person perspective, no tracking condition. To specifically test this hypothesis we analysed the flash-terminated trials more closely. As can be seen in Figure 7A the biological, 1^st^ person, no tracking condition shows the smallest value, i.e. the largest FLE (or smallest FLeadE). This was confirmed by a planned comparison that compared this condition to the corresponding symbolic condition, i.e. symbolic, 1^st^ person, no tracking (F(1,15) = 8.7, *p*<.01). In contrast, none of the other three simple comparisons between biological and symbolic stimuli (cf. Fig. 7A) reached significance (all p>.2). This pattern was further confirmed when we compared the biological, 1^st^ person, no tracking condition to all four symbolic conditions taken together (F(1,15) = 7.4, *p*<.02), and when we compared it to the remaining three biological conditions taken together (F(1,15) = 4.9, *p*<.05). Accordingly, the biological, 1^st^ person, no tracking condition is the main generator for the significant interaction between stimulus type and agency in an ANOVA calculated for flash-terminated trials only (F(1,15) = 5.5, *p<*.033, *η^2^_p_* = .276).

To complete the report of our results and to emphasize the differences between flash-terminated and continuous trials, we show the latter in Figure 7B. The interaction between stimulus type, perspective and agency depicted in Figure 6 is replicated here (F(1,15) = 5.2, *p*<.037, *η^2^_p_* = .259; all other *p*>.2). Most continuous conditions reveal negative values showing a general tendency for an FLE as reflected in the reported main effect of motion type.

## Discussion

First of all it is important to note that we replicated the classic pattern of results with the symbolic stimuli despite the fact that the velocity profile was not constant but identical to the profile of a biological movement (Fig. 2): We observed an FLE with continuous motion and no FLE (here expressed as a flash-lead effect) in the flash-terminated condition (Fig. 5). Although the effects are small this establishes an important ground truth that enables us to interpret the more interesting findings as true modulations of the FLE. To our knowledge this is also the first report of an FLE with a biological velocity profile (asymmetric acceleration and deceleration, Fig. 2). This motion velocity profile and the nature of our stimuli are also the most likely causes for the high interindividual variability in our data (Table 1), which, nevertheless allowed for a significant and meaningful pattern to emerge in our design with repeated measurement.

The most important outcome is that there was an interaction between perspective and agency for biological but not for symbolic stimuli. This shows that on average observers process the two stimulus types in different ways which cannot be attributed to low-level motion differences since the velocity profile was identical for the two conditions. We did predict an interaction between perspective and agency for biological but not for symbolic motion because the biological 1^st^ person perspective should generate the strongest motor resonance with the observer's MNS, potentially enhancing the FLE. However, this condition is also most susceptible to the illusion of voluntary control during tracking, which, according to Ichikawa and Masakura [16], reduces the FLE. Although the hypothesis that an illusion of control could be induced was rather speculative at this stage, it was based on observations [47], [48] that motor resonance in the MNS can induce an illusion of voluntary control and “movement ownership”, particularly when observed movements are visually compatible with the observer's limbs (i.e. in the 1^st^ person perspective, cf.[49]). Our data suggest that this was indeed the case for the biological 1^st^ person perspective when a tracking movement was required: The FLE was attenuated due to tracking in this condition conform to Ichikawa and Masakura's results in their ‘full’ and ‘partial control’ conditions, where they reported no FLE for concurrent arm movements with full or partial motion control. Their conclusion that observers' belief of controlling the motion on screen reduces the FLE is in full agreement with our result that tracking biological motion in the 1^st^ person perspective also diminishes the FLE (compare Fig. 6, left graph) because an illusion of control emerges. Hence, the observation that an illusion of control counteracts the FLE with symbolic motion [16] also holds true for biological motion.

Furthermore our data suggest that no illusion of control was induced with tracking in the biological 3^rd^ person perspective (Fig. 6). With a 3^rd^ person perspective motor resonance is generally weaker (cf. [49]) and an illusion of control therefore harder to establish. Another hint that the tracking movement in the biological 3^rd^ person perspective was not interpreted as voluntary motion control is that the pattern is similar for the symbolic conditions (Fig. 6, right graph). Although the tracking effect did not reach significance in a separate ANOVA for the symbolic stimuli (p>.1) it conforms numerically (Fig. 6, right graph) to Ichikawa and Masakura's result in their ‘no control’ condition, where they reported a larger FLE for concurrent arm movements without control over stimulus motion compared to a standard ‘no movement’ condition.

We have found compelling evidence that biological motion modulates the FLE in ways predicted by known characteristics of motor resonance in the MNS. In addition to the observed interaction between perspective and agency we also predicted that the condition that generates the strongest resonance–without inducing an illusion of control - could actually elicit an FLE in the flash-terminated trials by potentially strengthening the higher-level overshooting representation in competition with the veridical representation generated by the sudden offset of the stimulus (see Introduction). Again, this particular condition is the biological motion in the 1^st^ person perspective - without tracking. As predicted, this condition came closest in generating an FLE in the flash-terminated trials. This is in full agreement with the concept that action decoding in the MNS is predictive and thus further strengthens the idea of overshooting representations for moving stimuli.

While our results provide only indirect support for visual prediction, our findings directly impact on the “attentional shift” approach proposed by Baldo and Klein [55], [56]. In this account the flash diverts attention away from the moving stimulus and, hence, the stimulus cannot be bound to the accurate location and the emerging representation overshoots. In our paradigm we introduced agency and perspective with the latter only having a ‘meaning’ in the biological condition. The crucial point is that the stimuli in the 1^st^ and the 3^rd^ person perspective impose exactly the same binding requirements and the same attentional distraction by the flash. However, we found different patterns for the 1^st^ and 3^rd^ person perspective depending on agency for biological stimuli but not for symbolic stimuli. This interaction of agency and perspective as well as the difference between biological and symbolic stimuli are not adequately addressed by attention allocation and binding because the task requirements are identical for the two perspectives within each stimulus type. Although we fully agree that allocation of attention for efficient binding is an essential part of cognitive processing (see [57] for example) we conclude that it does not seem to be an essential factor for the FLE pattern we have obtained here.

We employed biological motion to specifically investigate the interaction between the visual and the sensorimotor systems since these stimuli are processed within both systems. A link between the two systems appears plausible because the FLE depends on the interaction between perspective and agency for biological motion, but not for symbolic stimuli. On the one hand this supports the notion that predictive representations in the sensorimotor system strongly influence the FLE, by either boosting or attenuating the effect. On the other hand this shows that prediction in the sensorimotor system alone cannot fully account for the FLE as our data show a different pattern in the symbolic condition. If sensorimotor predictive processing would account for the FLE then a similar pattern should be observed for biological and symbolic motion. We conclude that it is more likely that the FLE is generated within the early stages of the visual system and that the underlying mechanisms are predictive in the form of motion extrapolation. For this latter conclusion we cannot provide hard, ‘irrefutable’ evidence as other work has claimed (e.g. [6], [7], [10] see Introduction), but our predictions were based on the assumption that motor resonance in the MNS would help the overshooting representation against the offset representation. Indeed we have found evidence for this in the flash-terminated trials, where the condition with maximum motor resonance generated the strongest FLE. However, this FLE was still weaker than in the continuous motion condition, which, somewhat paradoxically, further underpins that motor resonance or sensorimotor prediction cannot fully cancel out the offset representation, hence, suggesting a specific interaction between the sensorimotor and the visual system. To conclude, our findings appear to be most compatible with the extended visual prediction notion [2] which assumes that extrapolated motion representations are generated within the visual system and can be modulated at various levels of processing by new visual input (e.g. offset signals) or by input from other systems (e.g. sensorimotor) that can boost or attenuate the overshooting representations in the form of biased neural competition [4].
